# Epitope Mapping of Anti‐Neurofascin 155 Antibody in a Large Cohort of Autoimmune Nodopathy Patients

**DOI:** 10.1002/acn3.70036

**Published:** 2025-03-25

**Authors:** Amina A. Abdelhadi, Hidenori Ogata, Xu Zhang, Takumi Tashiro, Ryo Yamasaki, Jun‐ichi Kira, Noriko Isobe

**Affiliations:** ^1^ Department of Neurology Neurological Institute, Graduate School of Medical Sciences, Kyushu University Fukuoka Japan; ^2^ Department of Medical Microbiology and Immunology, Faculty of Medicine Zagazig University Zagazig Egypt; ^3^ Translational Neuroscience Research Center, Graduate School of Medicine International University of Health and Welfare Okawa Japan

**Keywords:** autoantibody, autoimmune nodopathy, epitope, IgG4, neurofascin 155

## Abstract

**Objective:**

Autoimmune nodopathy (AN), a newly recognized disease entity, is an immune‐mediated polyneuropathy involving autoantibodies against cell adhesion molecules located in nodes of Ranvier and paranodal regions, such as neurofascin 186 (NF186) and neurofascin 155 (NF155). The present study aimed to identify the epitopes for autoantibodies against NF155 in a large cohort of Japanese patients with anti‐NF155 antibody‐positive (anti‐NF155+) AN.

**Methods:**

Human embryonic kidney 293 cells stably expressing NF155, NF186, or the third to fourth fibronectin type III domain region (Fn3‐Fn4) of NF155, as well as cells transiently expressing Fn3, Fn4, or the shorter Fn3‐Fn4 region of NF155, were developed. Western blotting and flow cytometric cell‐based assay (CBA) analyses were performed to determine the expression levels of the proteins and identify their target epitopes in serum samples from 100 IgG4 anti‐NF155+ patients, four non‐IgG4 anti‐NF155+ patients, and eight healthy controls.

**Results:**

The expression levels of NF186, NF155, Fn3‐Fn4 of NF155, and the other truncation variants of NF155 were confirmed by western blotting and flow cytometric CBA. Flow cytometric CBA analysis showed that the autoantibodies in all 104 anti‐NF155+ patients bound to Fn3‐Fn4. No autoantibodies reacted with NF186, Fn4, or shorter Fn3‐Fn4, although the autoantibodies in one IgG4 anti‐NF155+ patient (1.0%) recognized Fn3 in addition to Fn3‐Fn4. Western blotting analysis of representative samples generally reproduced the CBA results.

**Interpretation:**

The present study involving a large cohort of patients clarified that the primary epitope for anti‐NF155 antibodies is located in the Fn3‐Fn4 region, but not in the Fn3 or Fn4 domains alone.

## 1 Introduction

Chronic inflammatory demyelinating polyneuropathy (CIDP) is a heterogeneous acquired immune‐mediated neurological disorder that affects the peripheral nerves and nerve roots [1, 2]. On the basis of the clinical heterogeneity, distinct immunopathological mechanisms may underlie the various subtypes of CIDP, such as differences in immune responses or antigen specificities [3]. The pathogenesis of CIDP is primarily considered to involve T cells and macrophage‐mediated demyelination, although circulating humoral factors, such as autoantibodies, and activated plasma cells may also play roles [4, 5]. A recent study found that approximately 10% of CIDP patients possess autoantibodies against cell adhesion molecules located in nodes of Ranvier and paranodal regions, such as contactin‐1 (CNTN1), contactin‐associated protein‐1 (Caspr1), neurofascin 155 (NF155), and neurofascin 140/186 (NF140/186) [6]. In the latest update of the European Academy of Neurology/Peripheral Nerve Society CIDP diagnostic criteria, the distinctive characteristics of patients with these nodal/paranodal antibodies led to the establishment of a novel diagnostic category, termed autoimmune nodopathy (AN) [7]. Subclass analyses of the paranodal autoantibodies revealed a predominance of IgG4. However, IgG3 and IgG2 autoantibodies have also been detected, and these may be linked to rapid disease progression or a monophasic course [8].

**FIGURE 1:**
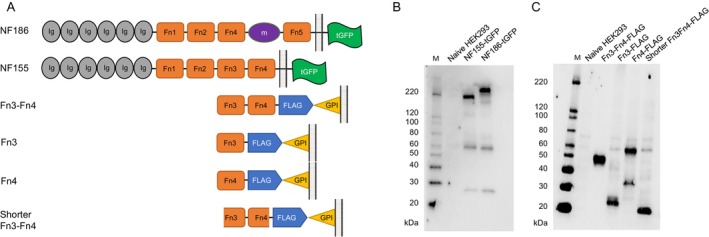
Domain structures and expression analysis of the NF isoforms and NF155 truncation variants used for epitope mapping. (A) The domain organizations of the NF isoforms (NF155 and NF186) and the truncated variants of NF155 (Fn3‐Fn4, Fn3, Fn4, and shorter Fn3‐Fn4) are shown. (B) In western blotting analyses, specific bands of NF155 and NF186, conjugated with tGFP, were obtained with an anti‐tGFP antibody. (C) The expression of each truncated variant conjugated with a FLAG tag and a GPI anchor was confirmed with an anti‐FLAG antibody. Two bands were observed for Fn4, the larger of which appeared to be a dimer. Fn3, third fibronectin type III domain; Fn4, fourth fibronectin type III domain; GPI, glycosylphosphatidylinositol; HEK293, human embryonic kidney 293 cells; M, molecular weight markers; m, mucin domain; NF, neurofascin; NF155, neurofascin 155; NF186, neurofascin 186; tGFP, turbo GFP.

Neurofascins (NFs) belong to the immunoglobulin superfamily, a large group of cell surface proteins with one or more extracellular immunoglobulin‐like (Ig) domains [9]. The major NF isoforms, NF186, NF155, and NF140, are generated and expressed in neural tissues by alternative splicing. Myelin terminal loops express NF155 in paranodes, where it interacts with axonal CNTN1 and Caspr1 to form a septal barrier that prevents the nodal complex from entering internodes [10]. NF186, a nodal NF isoform, has been implicated in dynamic synaptic stability, neuronal outgrowth, and sodium channel clustering [9]. NFs consist of six Ig domains, up to five fibronectin type III (Fn) domains, a transmembrane domain, and a short cytoplasmic domain [9]. The extracellular domains of NF155 and NF186 have the following differences: NF155 contains the first four Fn domains (Fn1‐Fn4), while NF186 lacks Fn3 and has a mucin domain between Fn4 and Fn5 (Figure 1A) [11]. Although the precise functions of the Fn domains in NF155 remain to be elucidated, Fn3 of NF155 was reported to promote neural cell adhesion and neurite outgrowth [12]. Therefore, this domain has the potential to bind to glial/axonal partners and play a role in paranode formation or stabilization [13].

AN patients with IgG4 anti‐NF155 antibodies exhibit unique features compared with autoantibody‐negative CIDP patients, including higher frequencies of human leukocyte antigen (HLA)‐DRB1*15 alleles, primary distal motor involvement, ataxia and low‐frequency tremor, and slower conduction delay on nerve conduction studies [14, 15]. Meanwhile, the IgG4 autoantibodies in these patients do not activate the complement pathway, mediate demyelination by stimulated macrophages, or induce inflammation; instead, they block functional protein–protein interactions at the paranodal axoglial junction, resulting in conduction breakdown [16, 17, 18, 19]. These observations are consistent with the electron microscopy findings for the sural nerves in patients with anti‐NF155 antibodies, which revealed detachment of the paranodal myelin terminal loops from the axolemma without inflammatory cell infiltration [20]. Furthermore, the IgG4 autoantibodies exhibited a pathogenic role in rodents, as their passive transfer reduced NF155 expression and prevented the formation of the NF155/Caspr1/CNTN1 complex [18]. In a recent multicenter international study, rituximab was effective in > 80% of anti‐NF155 antibody‐positive (anti‐NF155+) AN patients who showed a poor response to intravenous immunoglobulins [19]. Furthermore, rituximab was efficiently directed against CD20‐positive short‐lived plasma cells producing IgG4 and their CD20‐positive precursors [21], resulting in decreased B lymphocyte counts and serum IgG4 levels [21, 22, 23, 24].

Identifying epitopes recognized by autoantibodies can lead to a better understanding of disease mechanisms, more precise diagnosis, and the development of epitope‐specific therapeutic agents [25]. An epitope mapping study that evaluated 38 IgG4 anti‐NF155+ patients speculated that the Fn1‐Fn4 region contains the primary target epitope for the anti‐IgG4 NF155 antibodies [13], but it included some inconsistencies. Two other studies that investigated only four IgG4 anti‐NF155+ patients in total demonstrated that their sera reacted with the Fn3‐Fn4 region of NF155, but not with the Fn3 or Fn4 domains alone [8, 26]. Therefore, detailed epitope mapping of anti‐NF155 antibodies remains elusive.

In the present study, we aimed to identify the epitopes for anti‐NF155 antibodies in a large cohort of Japanese patients with anti‐NF155+ AN.

## 2 Methods

### 2.1 Subjects

The study enrolled 104 consecutive patients whose serum samples were found to be positive for total IgG anti‐NF155 antibodies by flow cytometric cell‐based assay (CBA) performed at Kyushu University. The samples were collected from the patients between 2008 and 2022. The 104 patients were subdivided into two groups based on their antibody subclass findings as follows: a group of 100 patients who were positive for IgG4 anti‐NF155 antibodies (IgG4 anti‐NF155+ patients) and a group of four patients who were positive for total IgG anti‐NF155 antibodies but negative for IgG4 anti‐NF155 antibodies (non‐IgG4 anti‐NF155+ patients). Sera from eight healthy subjects were also included as negative controls. The research protocols for the study were approved by the Kyushu University Ethics Committee. An opt‐out recruitment method was adopted for the study.

### 2.2 Development of Plasmid Vectors Encoding NF155 Truncation Variants

The NF155 fragments examined in the study were Fn3‐Fn4 (amino acid residues 836–1046), Fn3 (amino acid residues 836–942), Fn4 (amino acid residues 943–1046), and shorter Fn3‐Fn4 (amino acid residues 893–986) (Table 1 and Figure 1A). DNA fragments encoding these NF155 fragments were constructed by artificial DNA synthesis (FASMAC, Kanagawa, Japan) with a FLAG tag and glycosylphosphatidylinositol (GPI) anchor conjugation, and then cloned into pcDNA3.1/Zeo^(+)^ (Thermo Fisher Scientific, Waltham, MA).

**TABLE 1:** Summary of the domains cloned for expression in HEK293 cells.

Region in NF155	Protein fragment amino acid sequences defined by the NCBI	Protein fragment amino acid sequences in the present study
Fn3‐Fn4	Ala839‐Ser1034	Pro836‐Ala1046
Fn3	Ala839‐Thr938	Pro836‐Val942
Fn4	Pro943‐Ser1034	Pro943‐Ala1046
Shorter Fn3‐Fn4		Gln893‐Ile986

Abbreviations: Fn3, third fibronectin type III domain; Fn4, fourth fibronectin type III domain; HEK293, human embryonic kidney 293 cells; NCBI, National Center for Biotechnology Information; NF155, neurofascin 155.

### 2.3 Development of Stable Cell Lines Expressing NF186, NF155, and Fn3‐Fn4 of NF155

In addition to human embryonic kidney 293 (HEK293) cell lines stably expressing NF155 or NF186 with turbo GFP (tGFP) established in a previous study [27], HEK293 cells stably expressing Fn3‐Fn4‐FLAG‐GPI anchor were newly developed for the present study. Briefly, the vector was transfected into naive HEK293 cells using FuGENE 6 Transfection Reagent (Promega Corporation, Madison, WI) in accordance with the manufacturer's instructions. The transfected cells were cultured in selection medium containing G418 (Thermo Fisher Scientific). When single colonies of adequate size for clonal expansion had formed, individual colonies were picked up using a sterile cloning cylinder and scaled up to larger volumes.

### 2.4 Transient Expression of Various NF155 Truncation Variants in HEK293 Cells

The epitope mapping vectors encoding Fn3, Fn4, or shorter Fn3‐Fn4 conjugated with a FLAG tag and a GPI anchor were separately transfected into HEK293 cells using FuGENE 6 Transfection Reagent (Promega Corporation) in accordance with the manufacturer's instructions. After incubation at 37°C for 24–48 h, the cells were processed for experiments.

### 2.5 Western Blot Analysis

Cells stably expressing NF155, NF186, or Fn3‐Fn4 and cells transiently expressing Fn3, Fn4, or shorter Fn3‐Fn4 were lysed with radioimmunoprecipitation buffer containing a protease inhibitor cocktail and 0.5% sodium dodecyl sulfate (SDS) (Nacalai Tesque, Kyoto, Japan). Cell lysates from naive HEK293 cells were used as a negative control.

The protein concentrations in the cell lysates were measured using a BCA Protein Assay Kit (Thermo Fisher Scientific). The cell lysates were then mixed with Laemmli buffer and heated at 95°C for 3 min. Subsequently, the samples were electrophoresed in 7.5% mini PROTEAN precast gradient gels (Bio‐Rad, Hercules, CA), transferred onto polyvinylidene fluoride membranes, blocked with 3% skim milk, and incubated with patient sera (1:1000 dilution) or the following primary antibodies: mouse anti‐FLAG antibody (FUJIFILM Wako Chemicals, Osaka, Japan; 1:2000 dilution); rabbit anti‐tGFP antibody (Evrogen, Moscow, Russia; 1:5000 dilution). NF155‐tGFP and NF186‐tGFP were specifically detected using the anti‐tGFP antibody, while Fn3, Fn4, Fn3‐Fn4, and shorter Fn3‐Fn4‐FLAG‐GPI anchor were detected using the anti‐FLAG antibody. After washing, the membranes were incubated with secondary antibodies (1:20,000 dilution) for 1 h at room temperature. The following secondary antibodies were used: horseradish peroxidase (HRP)‐conjugated goat antihuman IgG Fc antibody (Abcam, Cambridge, UK); HRP‐conjugated horse anti‐mouse IgG antibody (Vector Laboratories, Burlingame, CA); HRP‐conjugated goat anti‐rabbit IgG antibody (Vector Laboratories). After treatment with ECL Prime Western Blotting Detection Reagent (GE Healthcare, Chicago, IL), the membrane‐bound antibodies were identified and analyzed using a ChemiDoc XRS System (Bio‐Rad).

### 2.6 Cell‐Based Assay of Transiently Transfected Cells and Stable Cell Lines by Flow Cytometry

The cell lines stably expressing NF155, NF186, or Fn3‐Fn4 were separately mixed with naive HEK293 cells and resuspended in Dulbecco's modified Eagle's medium containing 1% fetal bovine serum and 1 mmol/L ethylenediaminetetraacetic acid (EDTA) (FCM buffer) at a concentration of 1.0 × 10 [6] cells/mL. At 24–48 h after transfection with the vectors encoding Fn3, Fn4, or shorter Fn3‐Fn4, the cells were resuspended in FCM buffer at a concentration of 1.0 × 10 [6] cells/mL. Serum samples were mixed with 50 μL of cell‐containing solution at a final dilution of 1:200. After incubation at room temperature for 90 min on a shaker, the cells were washed. Bound autoantibodies were detected using an Alexa Fluor 647‐conjugated antihuman IgG antibody (Thermo Fisher Scientific; 1:500 dilution). After incubation at room temperature for 40 min on a shaker, the cells were washed and resuspended in 100 μL of phosphate‐buffered saline containing 5 mmol/L EDTA and analyzed using a MACSQuant Analyzer (Miltenyi Biotec, Bergisch Gladbach, Germany).

As a positive control, purified IgG from one IgG4 anti‐NF155+ patient was examined. Expression of NF186 was confirmed using a rabbit anti‐NF186 antibody (Chemicon International, Temecula, CA; 1:500 dilution) and an Alexa Fluor 647‐conjugated anti‐rabbit IgG antibody (Thermo Fisher Scientific; 1:500 dilution). An Alexa Fluor 647‐conjugated anti‐FLAG antibody (MBL, Tokyo, Japan; 1:500 dilution) was used to detect Fn3, Fn4, Fn3‐Fn4, and shorter Fn3‐Fn4.

**FIGURE 2:**
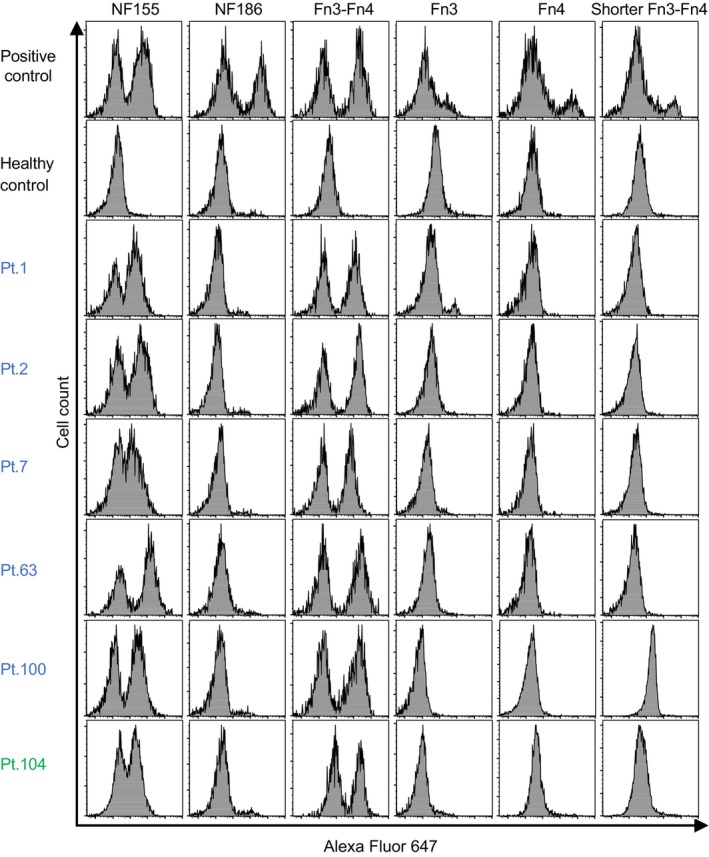
Flow cytometric CBA for the reactivities of anti‐NF155+ patient sera against specific NF isoforms and truncated variants of NF155. The *X*‐axis and *Y*‐axis in each histogram indicate the fluorescence intensity of Alexa Fluor 647 and the cell count, respectively. Rows 1 and 2 show representative histograms of positive and negative controls, respectively. The flow cytometric CBA results for representative serum samples from five IgG4 anti‐NF155+ patients (Pts. 1, 2, 7, 63, 100; blue) and one non‐IgG4 anti‐NF155+ patient (Pt. 104; green) are shown in rows 3–8. All serum samples from the six patients were reactive toward NF155 but not NF186. Epitope analysis using the NF155 truncation variants showed that the reactivity against Fn3‐Fn4 was present in all serum samples positive for full‐length NF155. None of the patient sera were reactive toward Fn4 and shorter Fn3‐Fn4. The serum sample from only one IgG4 anti‐NF155+ patient (Pt. 1) reacted with Fn3. Fn3, third fibronectin type III domain; Fn4, fourth fibronectin type III domain; NF, neurofascin; NF155, neurofascin 155; NF186, neurofascin 186; Pt., Patient; tGFP, turbo GFP.

FlowJo software version 10.8.1 (Becton Dickinson, San Jose, CA) was used to analyze the flow cytometry data and prepare the figures. Flow cytometric histograms were created to evaluate the relative fluorescence intensities of Alexa Fluor 647 (Figure 2). As positive controls, purified IgG from one IgG4 anti‐NF155+ patient, rabbit anti‐NF186 antibody, and anti‐FLAG antibody were used to detect NF155, NF186, and the truncated variants of NF155, respectively. Serum from a healthy control was adopted as a negative control. Positive flow cytometry results were defined as histograms exhibiting two peaks representing cells with and without the target proteins captured by the autoantibodies. Histograms showing only one peak were defined as negative flow cytometry results.

## 3 Results

### 3.1 Demographic Data, Clinical Characteristics, and Laboratory Data for the Anti‐NF155+ Patients

The demographic, clinical, and laboratory characteristics for the 100 IgG4 anti‐NF155+ patients and four non‐IgG4 anti‐NF155+ patients are summarized in Table 2. Their mean ages at onset were early to mid‐30s, relatively younger than those of general CIDP patients. The symptoms and neurological findings were usually symmetrical, and > 40% of the patients predominantly exhibited distal manifestations. All patients met definite European Federation of Neurological Societies/Peripheral Nerve Society electrophysiological criteria for CIDP except for one IgG4 anti‐NF155+ patient, in whom compound muscle action potentials were not evoked in any nerves. The mean CSF protein levels were highly elevated in both groups.

**TABLE 2:** Demographic and laboratory parameters in the IgG4 anti‐NF155+ and non‐IgG4 anti‐NF155+ AN patients.

	IgG4 anti‐NF155+ AN (*N* = 100)	Non‐IgG4 anti‐NF155+ AN (*N* = 4)
Demographic data		
Sex ratio, male:female	82:18	2:2
Age at onset (years), mean ± SD	33.0 ± 17.2	35.0 ± 16.3
Age at sample collection (years), mean ± SD^a^	37.8 ± 17.9	44.3 ± 10.4
Disease duration (months), median (IQR)^a^	15 (6.5, 64)	107 (22, 212.25)
Clinical subtypes^a^		
Typical, *n* (%)	52 (53)	2 (50)
DADS, *n* (%)	42 (43)	2 (50)
MADSAM, *n* (%)	0	0
Pure sensory, *n* (%)	4 (4)	0
Definite electrophysiological criteria, *n* (%)	99 (99)	4 (100)
CSF protein amounts (mg/dL), mean ± SD	342 ± 186	273 ± 128

Abbreviations: AN, autoimmune nodopathy; CSF, cerebrospinal fluid; DADS, distal acquired demyelinating symmetric neuropathy; IQR, interquartile range; MADSAM, multifocal acquired demyelinating sensory and motor neuropathy; *N*, number of patients collated; *n*, number of positive patients; NF155, neurofascin 155; SD, standard deviation.

^a^ Data for two patients were missing among the IgG4 anti‐NF155+ AN patients.

### 3.2 Confirmation of Expression of NF155, NF186, and NF155 Domains in HEK293 Cells

The expression of the recombinant proteins in the cell lines was confirmed by western blotting (Figure 1B,C). Two bands were noted for Fn4, suggesting the presence of a dimer (Figure 1C).

The flow cytometric CBA also confirmed the protein expression. The purified IgG from one IgG4 anti‐NF155+ patient, anti‐NF186 antibody, and anti‐FLAG antibody stained cells expressing NF155, NF186, and truncation variants of NF155, respectively, and showed two apparent peaks in the histograms (Figure 2).

### 3.3 Epitope Mapping of NF155 Antibodies by Flow Cytometric CBA

To determine the epitopes for the anti‐NF155 antibodies in this large cohort of anti‐NF155+ patients, the flow cytometric CBA was adopted (Figure S1). Representative results are shown in Figure 2, and a data summary is presented in Table 3. None of the serum samples from the IgG4 anti‐NF155+ patients reacted with NF186 (Table 3 and Figure 2). Surprisingly, all 100 of these serum samples bound to Fn3‐Fn4. However, none of the 100 samples reacted with Fn3 or Fn4 alone, except for one sample that also bound to Fn3. These findings led us to hypothesize that the main epitopes existed in the transition region between Fn3 and Fn4. However, none of the samples from the IgG4 anti‐NF155+ patients reacted with shorter Fn3‐Fn4, in which both sides of Fn3‐Fn4 were truncated. As expected, none of the eight healthy control serum samples reacted with either the NF isoforms (NF155 and NF186) or the truncated variants of NF155 (Fn3‐Fn4, Fn3, Fn4, and shorter Fn3‐Fn4) (Table 3, Figure 2, and Figure S1). When the four non‐IgG4 anti‐NF155+ patients were evaluated, all four serum samples reacted with Fn3‐Fn4, while none of the samples reacted with NF186 or the other truncation variants of NF155 (Table 3 and Figure 2), demonstrating similar characteristics to the samples from the IgG4 anti‐NF155+ patients.

**TABLE 3:** Summary of the results obtained by flow cytometric CBA among the anti‐NF155+ AN patients.

	NF186, *n* (%)	NF155, *n* (%)	Fn3‐Fn4, *n* (%)	Fn3, *n* (%)	Fn4, *n* (%)	Shorter Fn3‐Fn4, *n* (%)
IgG4 anti‐NF155+ AN *N* = 100	0 (0)	100 (100)	100 (100)	1 (1.0)	0 (0)	0 (0)
Non‐IgG4 anti‐NF155+ AN *N* = 4	0 (0)	4 (100)	4 (100)	0 (0)	0 (0)	0 (0)
Healthy controls *N* = 8	0 (0)	0 (0)	0 (0)	0 (0)	0 (0)	0 (0)

Abbreviations: AN, autoimmune nodopathy; CBA, cell‐based assay; Fn3, third fibronectin type III domain; Fn4, fourth fibronectin type III domain; *N*, number of patients collated; *n*, number of positive patients; NF155, neurofascin 155; NF186, neurofascin 186.

### 3.4 Validation of the Epitope Mapping for the Anti‐NF155 Antibodies by Western Blotting

**FIGURE 3:**
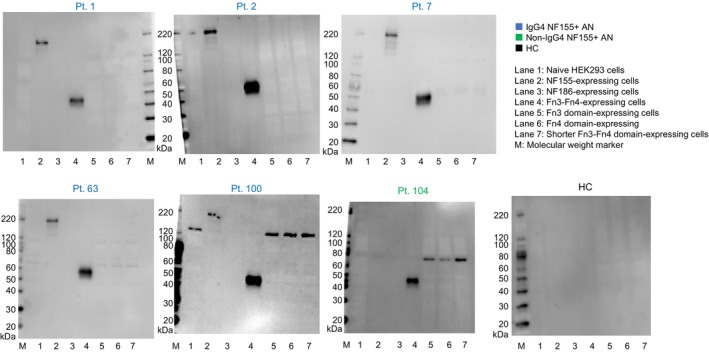
Immunoblotting analysis to validate the reactivities of anti‐NF155+ patient sera against specific NF isoforms and truncated variants of NF155. Serum samples from five IgG4 anti‐NF155+ patients (Pts. 1, 2, 7, 63, 100; blue), one non‐IgG4 anti‐NF155+ patient (Pt. 104; green), and one healthy control were selected as sources for the primary antibodies. All serum samples from the IgG4 anti‐NF155+ patients were reactive toward NF155‐tGFP (lane 2) and Fn3‐Fn4‐FLAG‐GPI (lane 4). The serum sample from the one non‐IgG4 anti‐NF155+ patient reacted with Fn3‐Fn4 only (lane 4). The serum sample from the healthy control showed no reactivity toward the NF isoforms or the truncated variants of NF155. Fn3, third fibronectin type III domain; Fn4, fourth fibronectin type III domain; GPI, glycosylphosphatidylinositol; HC, healthy control; HEK293, human embryonic kidney 293 cells; M, molecular weight markers; NF, neurofascin; NF155, neurofascin 155; NF186, neurofascin 186; Pt., Patient; tGFP, turbo GFP.

Representative serum samples from five IgG4 anti‐NF155+ patients, one non‐IgG4 anti‐NF155+ patient, and one healthy control were further selected for western blotting analysis. The sera from all six anti‐NF155+ patients (Pts. 1, 2, 7, 63, 100, and 104) showed specific bands in the lanes containing full‐length NF155 and Fn3‐Fn4, although the serum sample from the non‐IgG4 anti‐NF155+ patient (Pt. 104) had a very weak reaction with full‐length NF155 (Figure 3). The sample that was reactive toward Fn3 in the flow cytometric CBA did not show any reactivity to Fn3 in the western blotting analysis. The healthy control sample did not react with any of the proteins (Figure 3).

## 4 Discussion

This epitope mapping study for anti‐NF155 antibodies utilizing sera from 104 anti‐NF155+ patients produced the following findings: (1) none of the anti‐NF155+ patient sera were reactive toward NF186; (2) all of the anti‐NF155+ patient sera bound to Fn3‐Fn4; (3) none of the anti‐NF155+ patient sera were reactive toward Fn4 or shorter Fn3‐Fn4; (4) the serum of only one IgG4 anti‐NF155+ patient bound to Fn3; (5) sera from four non‐IgG4 anti‐NF155+ AN patients also reacted with Fn3‐Fn4.

NF155 and NF186 have very similar structures, with the only differences being that NF155 lacks the mucin domain and Fn5, while NF186 lacks Fn3 [28]. The Fn3 domain is known to interact with integrins and has been implicated in the initial axoglial interaction through its arginine, glycine, and aspartic acid (RGD) motif [29]. This RGD sequence within NF155 was also reported to facilitate neurite development and neuronal cell spreading [12]. Given our findings that IgG4 anti‐NF155+ patient sera were not reactive toward NF186, we hypothesized that the antigenic epitopes for anti‐NF155 antibodies are located around the extracellular Fn3 domain unique to NF155, consistent with previous reports [8, 26, 30].

With confirmed expression of NF155, NF186, and several truncated variants of NF155 on the surface of HEK293 cells by western blotting and flow cytometric CBA analyses, the flow cytometric CBA using live human cell lines, which provides more accurate results than a fixed CBA [31, 32], clarified that all examined sera from IgG4 anti‐NF155+ patients were reactive toward Fn3‐Fn4, indicating that this region contains the pivotal epitope for IgG4 anti‐NF155 antibodies. We further investigated the binding capacity of IgG4 anti‐NF155 antibodies against Fn3 or Fn4 alone to identify more specific epitopes. However, none of the sera, except for one sample from an IgG4 anti‐NF155+ patient, reacted with Fn3. Although we tested the reactivity of the IgG4 anti‐NF155+ patient sera against shorter Fn3‐Fn4, which contained the transition part between Fn3 and Fn4, none of the samples reacted with this truncated variant. Finally, we confirmed compatible results between the western blotting and flow cytometric CBA analyses using representative samples. Taken together, IgG4 anti‐NF155 antibodies are likely to recognize conformational epitopes in Fn3‐Fn4, but not in Fn3 or Fn4 alone.

To our knowledge, only a few reports have mentioned epitopes for anti‐NF155 antibodies [12, 18, 29]. One epitope mapping study found that the Fn1‐Fn4 region of NF155 contained the main target epitope for IgG4 NF155 antibodies in 30 of 38 patients (79%) because their sera did not bind to NF155 that lacked this region [13]. However, the study also found that the autoantibodies from 16 of 38 patients (42%) did not bind to Fn1‐Fn4 [13]. Ng et al. [26] mapped the domains recognized by the autoantibodies of two IgG4 anti‐NF155+ CIDP patients using truncation variants of NF155 and NF186, and found that the autoantibodies from both patients bound to Fn3‐Fn4, but not Fn3 or Fn4 alone. Similarly, Stengel and colleagues reported that the sera from two other IgG4 anti‐NF155+ patients were immunoreactive toward Fn3‐Fn4 [8]. The findings of these studies were generally in accordance with the present results despite their limited numbers of cases. Our study with a much larger number of patients proved that the specific epitopes for IgG4 anti‐NF155 antibodies are generally located in Fn3‐Fn4.

Two specific *HLA* class II alleles, HLA‐DRB1*15:01 and HLA‐DRB1*15:02, were found to be present in > 90% of IgG4 anti‐NF155+ patients [15, 33], representing a significantly higher proportion compared with the general population. Class II HLA molecules on antigen‐presenting cells play a role in showing antigen peptides to CD4+ T cells with specific T‐cell receptors [34]. Because HLA‐DRB1*15:01 and HLA‐DRB1*15:02 have only one difference at the 86th amino acid, located in pocket 1 of the peptide‐binding groove [35, 36], both of these HLA class II molecules could present common NF155 peptides to CD4+ T cells [1, 33]. Because B cells, in addition to their function in antibody production, also express high levels of MHC class II molecules and can present antigens [37], B cells with both HLA‐DRB1*15 and B cell receptors against epitopes in Fn3‐Fn4 may provide common peptides to specific CD4+ T cells.

This study also evaluated four non‐IgG4 anti‐NF155+ patients. Although it remains uncertain whether IgG4 anti‐NF155+ and non‐IgG4 anti‐NF155+ AN are identical in terms of the roles of autoantibodies and their pathomechanisms, the findings for these patients were similar to those for IgG4 anti‐NF155+ patients. Further studies are needed to clarify this point.

The present study had some limitations. First, the Fn3‐Fn4 region that we identified as containing the specific epitope for anti‐NF155 antibodies still contains approximately 200 amino acids. Although our results suggest that the anti‐NF155 antibodies recognize a conformational structure within Fn3‐Fn4, western blotting revealed that all patient samples were reactive toward processed full‐length NF155 and Fn3‐Fn4 after SDS‐polyacrylamide gel electrophoresis. Because some β‐sheet proteins, including Fn domains, were reported to show resistance against SDS denaturation [38], the conformational epitopes in Fn3‐Fn4 may be maintained under the experimental conditions used in this study. Further approaches to narrow down the target epitopes will be needed in future studies. Second, we did not evaluate the effects of the sugar chains in glycosylated NF155 and its different domain constructs on the autoantibody binding. The sugar chains in NF155 are located in Fn4 on two amino acids (Asn973 and Asn988), with no chains found in Fn3 [39]. N‐glycosylations are essential for the construction of the paranodal complex [40]. Notably, the binding of NF155 to CNTN1 requires N‐glycosylation, and in turn, NF155 binds to glycosylated CNTN1 using mannose‐rich oligosaccharides [41]. Chataigner et al. [42] reported that either CNTN1 or NF155, but not both, is rich in mannose glycans and promotes heterophilic cell–cell interactions. In a previous study that investigated the contribution of NF155 glycosylation [13], deglycosylation by either N‐glycosidase F or tunicamycin extinguished the autoantibody binding to NF155. Labasque et al. [43] determined that the immunoreactivity was directed against the Ig domains of CNTN1 and was N‐glycan‐dependent. These types of analyses will be considered for our future studies.

In conclusion, this epitope mapping study involving a large cohort of Japanese anti‐NF155+ AN patients has provided detailed information about the specificity of anti‐NF155 antibodies. We propose that Fn3‐Fn4, but not Fn3 or Fn4 alone, constitutes the primary epitope for anti‐NF155 antibodies. This determination allows us to understand the pathogenesis of the disorder more deeply and to develop innovative epitope‐specific therapeutic strategies, such as decoy antigen therapies and more specific diagnostic procedures. Further efforts are warranted to determine more specific epitopes for these autoantibodies.

## Author Contributions

A.A., H.O., X.Z., T.T., R.Y., J.K., and N.I. designed and conceptualized the study, collected the data, analyzed the data, and drafted the manuscript.

## Ethics Statement

The research protocols for the study were approved by the Kyushu University Ethics Committee.

## Consent

An opt‐out recruitment method was adopted for the present study.

## Conflicts of Interest

The authors declare no conflicts of interest.

## Supporting information


Figure S1.

## Data Availability

The data that support the findings of this study are available from the corresponding author upon reasonable request.
