# Supplementary material for: Epitope Mapping of Anti‐Neurofascin 155 Antibody in a Large Cohort of Autoimmune Nodopathy Patients
Source: Ann Clin Transl Neurol. 2025 Mar 25;12(5):1034–42. doi: 10.1002/acn3.70036 (PMC12093329; doi:10.1002/acn3.70036)
Supplement: Supplementary file 1 — Figure S1. [file ACN3-12-1034-s001.pdf]

Figure S1 Whole data of flow cytometric CBA for the reactivities of anti-NF155+ patient sera against specific NF isoforms and truncated variants of NF155

The X-axis and Y-axis in each histogram indicate the fluorescence intensity of Alexa Fluor 647 and the cell count, respectively. The flow cytometric CBA results for 100 IgG4 anti-NF155+ patients (Pts. 1 to 100) and four non-IgG4 anti-NF155+ patient (Pts. 101 to 104) are shown.

CBA, cell-based assay; Fn3-Fn4, third and fourth fibronectin type III domains; Fn3, third fibronectin type III domain; Fn4, fourth fibronectin type III domain; NF, neurofascin; NF155, neurofascin 155; NF186, neurofascin 186; Pt., Patient.

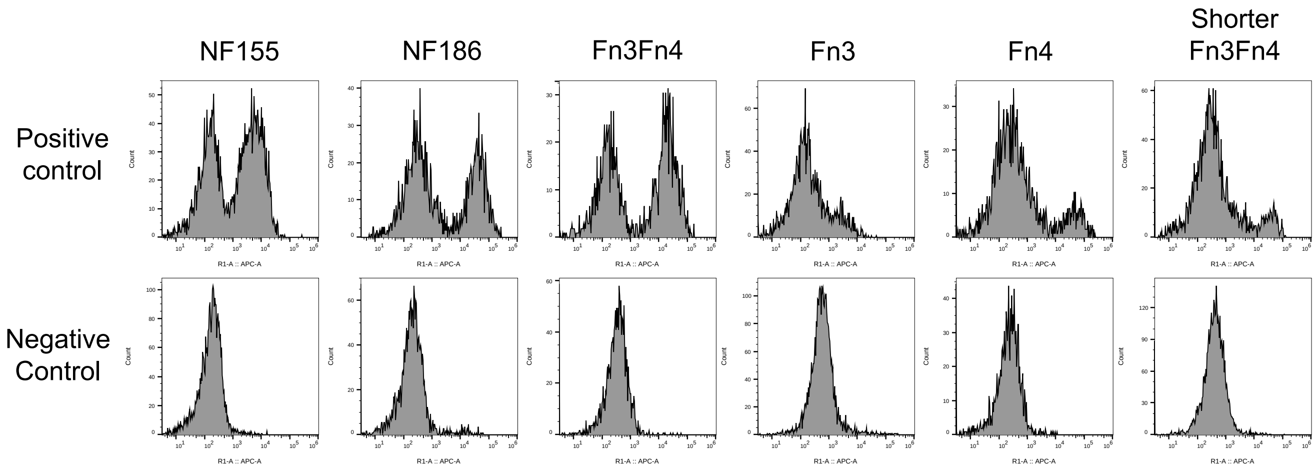
NF155
NF186
Fn3Fn4
Fn3
Fn4
Shorter Fn3Fn4
Positive control
Negative Control
Count
R1-A :: APC-A

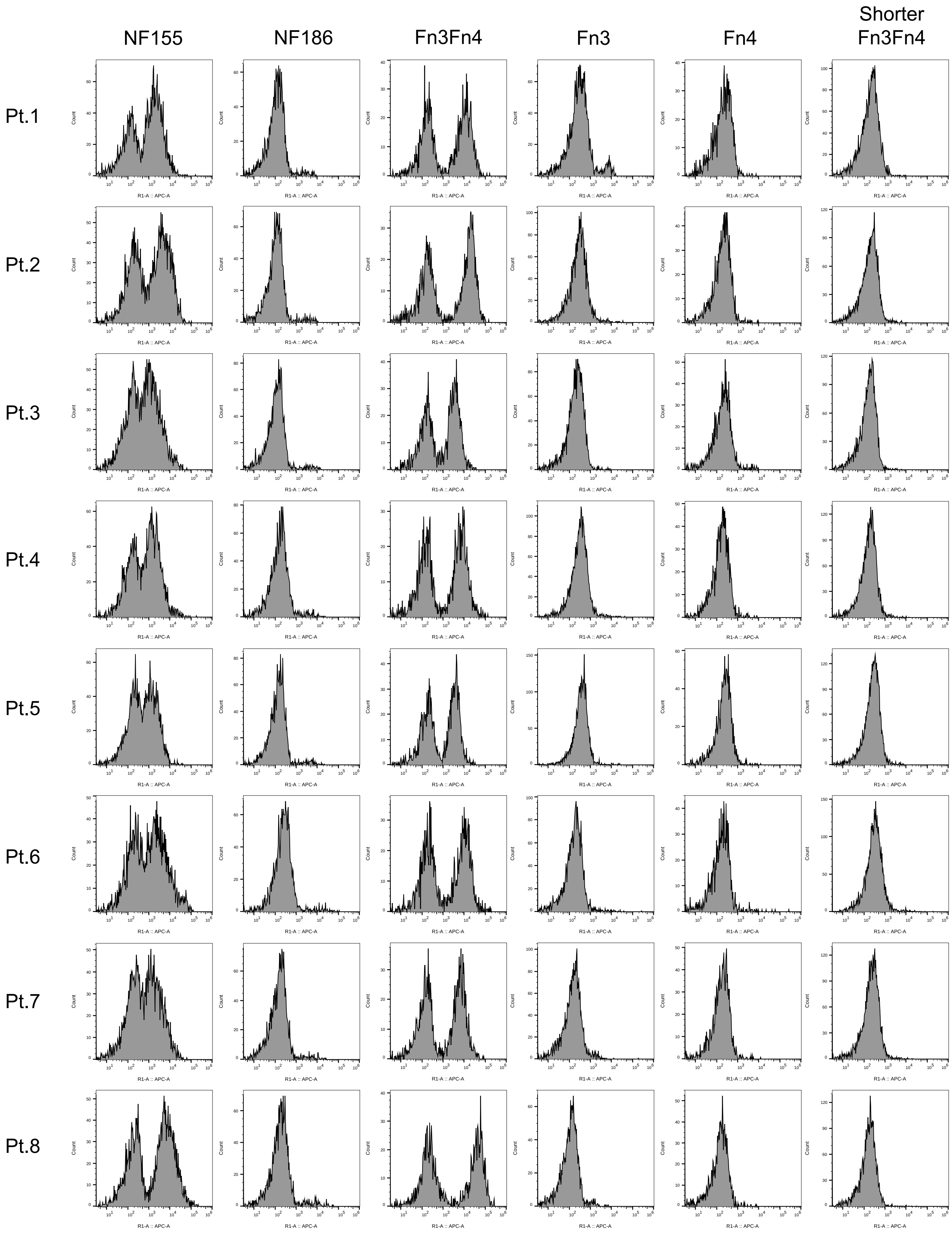
NF155
NF186
Fn3Fn4
Fn3
Fn4
Shorter Fn3Fn4
Pt.1
Pt.2
Pt.3
Pt.4
Pt.5
Pt.6
Pt.7
Pt.8
Count
R1-A :: APC-A

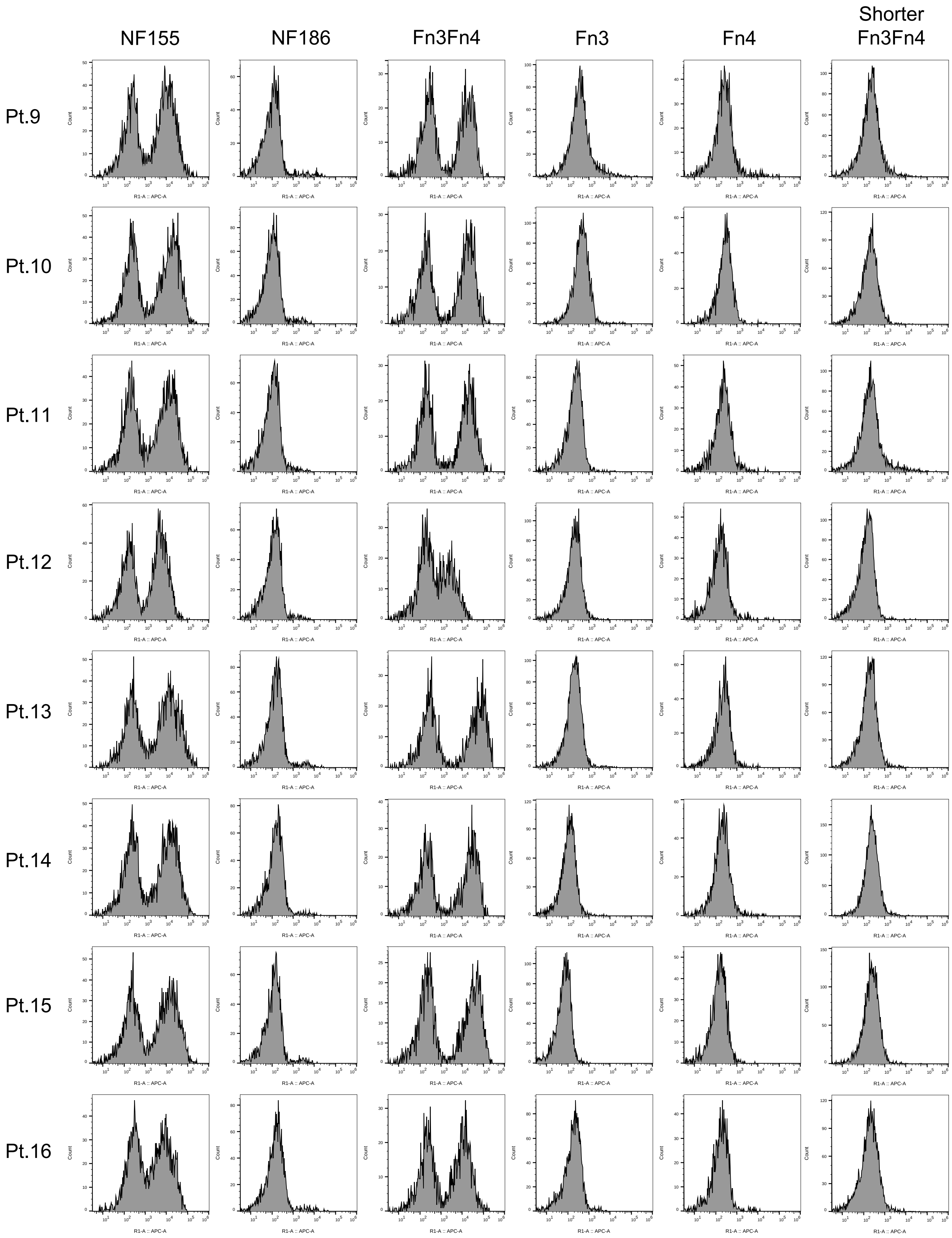
NF155
NF186
Fn3Fn4
Fn3
Fn4
Shorter Fn3Fn4
Pt.9
Pt.10
Pt.11
Pt.12
Pt.13
Pt.14
Pt.15
Pt.16
Count
R1-A :: APC-A

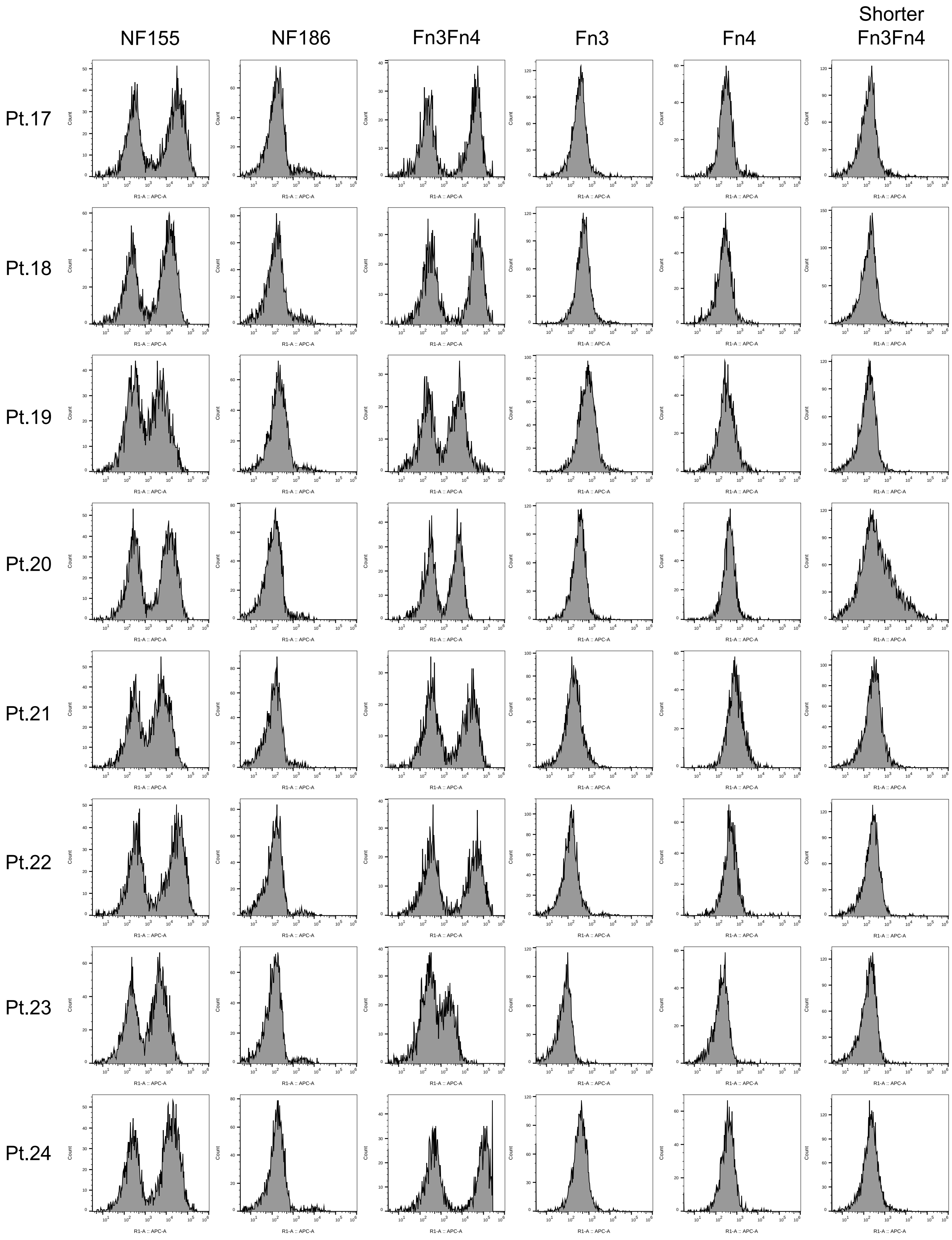
NF155
NF186
Fn3Fn4
Fn3
Fn4
Shorter Fn3Fn4
Pt.17
Pt.18
Pt.19
Pt.20
Pt.21
Pt.22
Pt.23
Pt.24
Count
R1-A :: APC-A

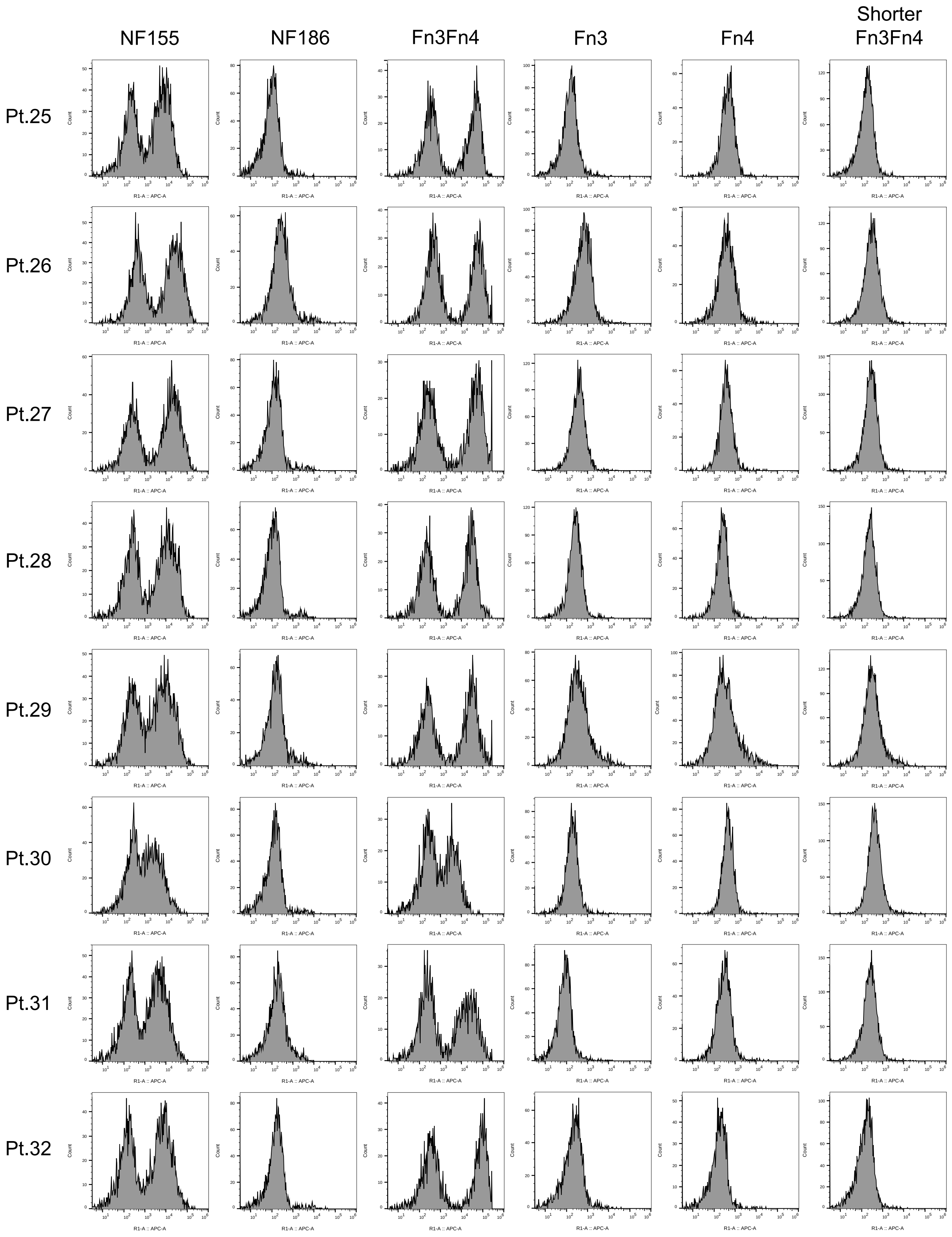
NF155
NF186
Fn3Fn4
Fn3
Fn4
Shorter Fn3Fn4
Pt.25
Pt.26
Pt.27
Pt.28
Pt.29
Pt.30
Pt.31
Pt.32
Count
R1-A :: APC-A

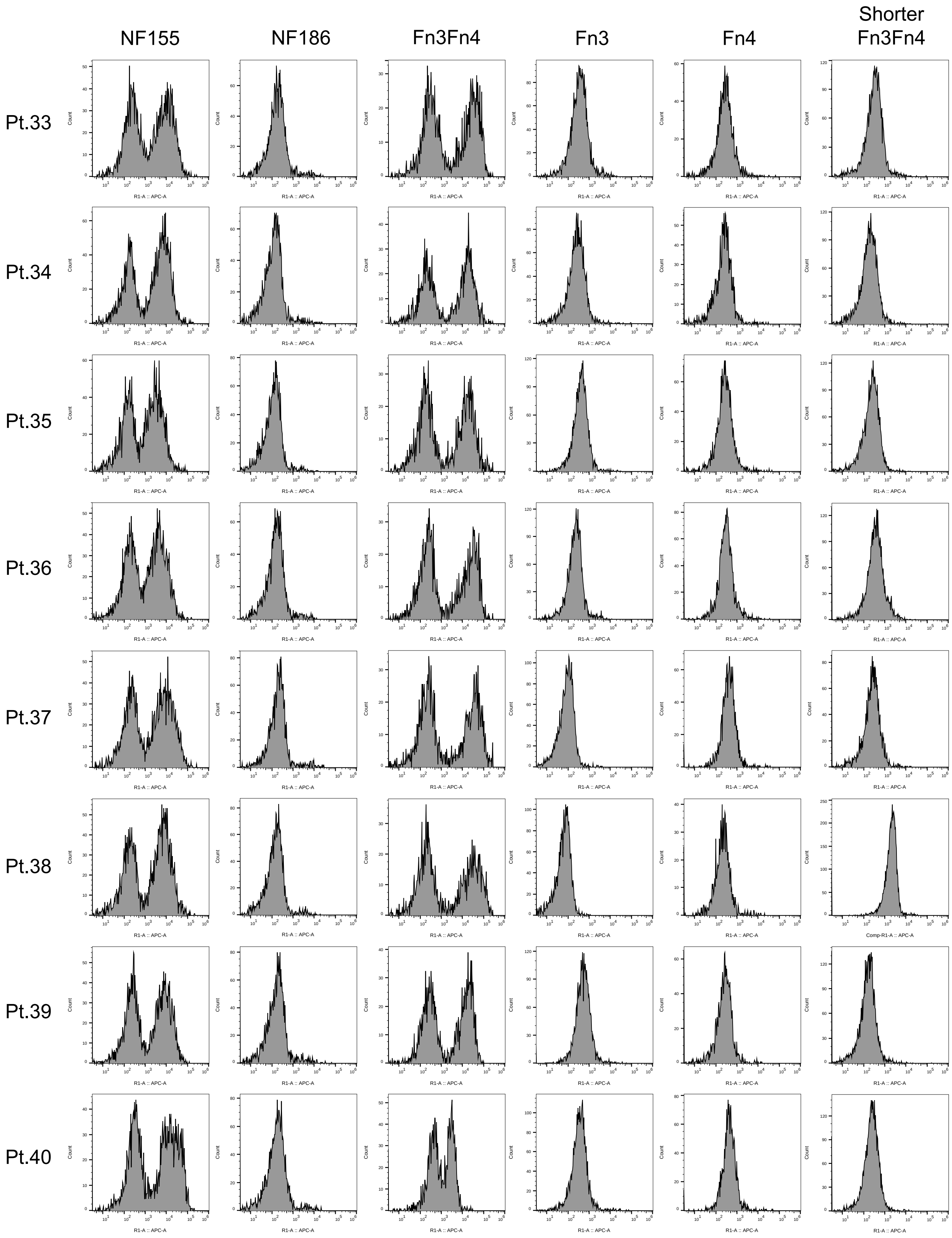

NF155
NF186
Fn3Fn4
Fn3
Fn4
Shorter Fn3Fn4
Pt.33
Pt.34
Pt.35
Pt.36
Pt.37
Pt.38
Pt.39
Pt.40
Count
R1-A :: APC-A
Comp-R1-A :: APC-A

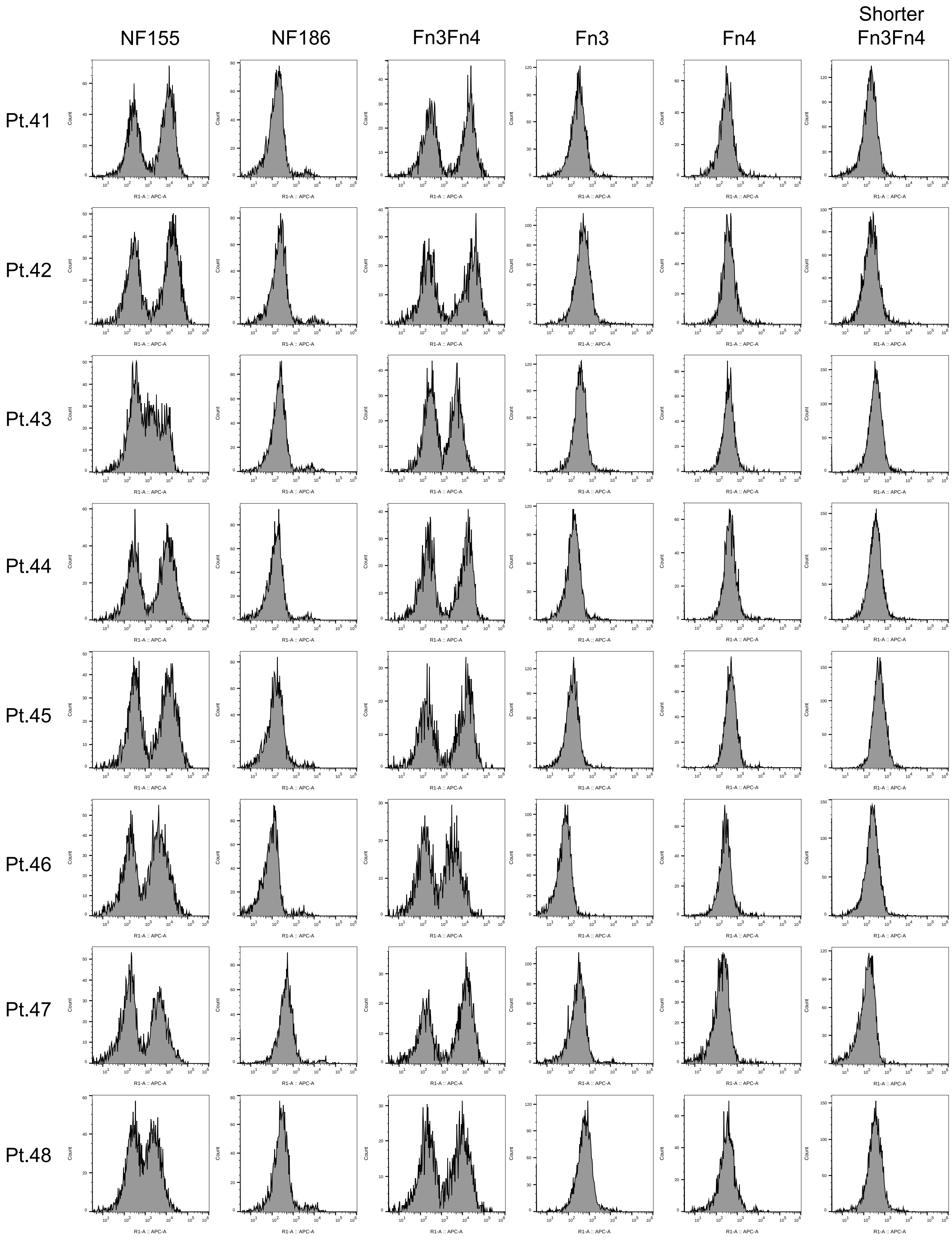

NF155
NF186
Fn3Fn4
Fn3
Fn4
Shorter Fn3Fn4
Pt.41
Pt.42
Pt.43
Pt.44
Pt.45
Pt.46
Pt.47
Pt.48
Count
R1-A :: APC-A

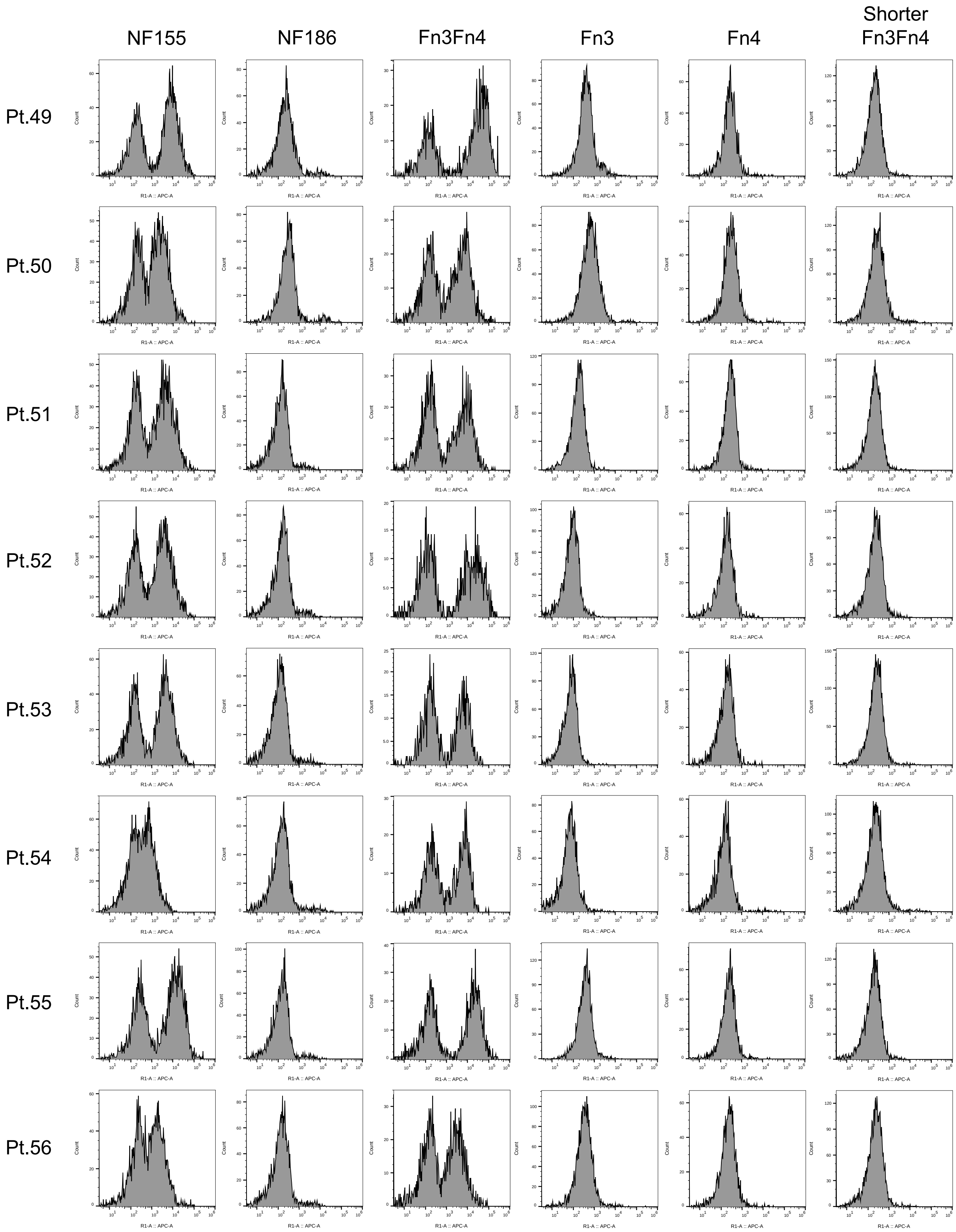
NF155
NF186
Fn3Fn4
Fn3
Fn4
Shorter Fn3Fn4
Pt.49
Pt.50
Pt.51
Pt.52
Pt.53
Pt.54
Pt.55
Pt.56
Count
R1-A :: APC-A

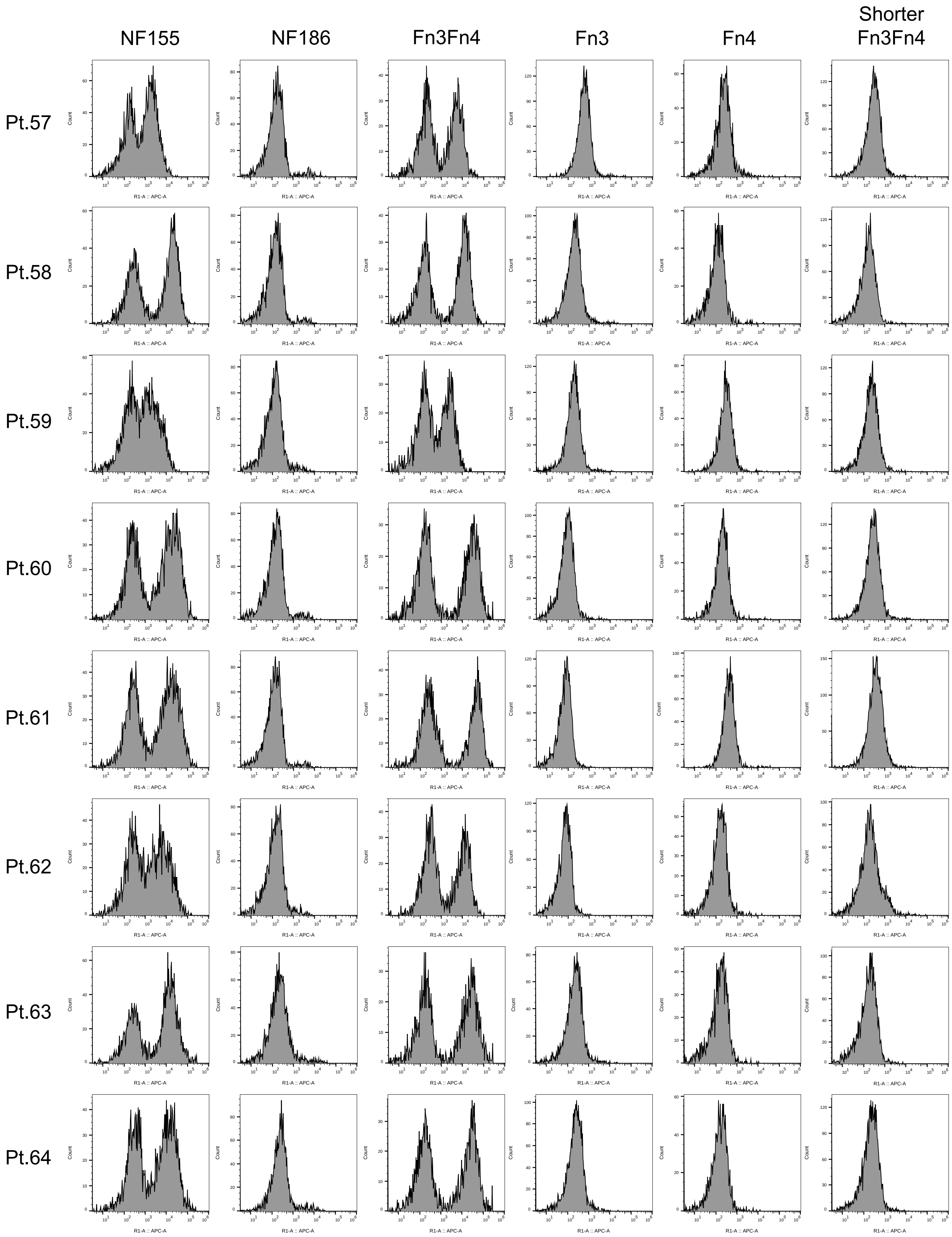
NF155
NF186
Fn3Fn4
Fn3
Fn4
Shorter Fn3Fn4
Pt.57
Pt.58
Pt.59
Pt.60
Pt.61
Pt.62
Pt.63
Pt.64
Count
R1-A :: APC-A

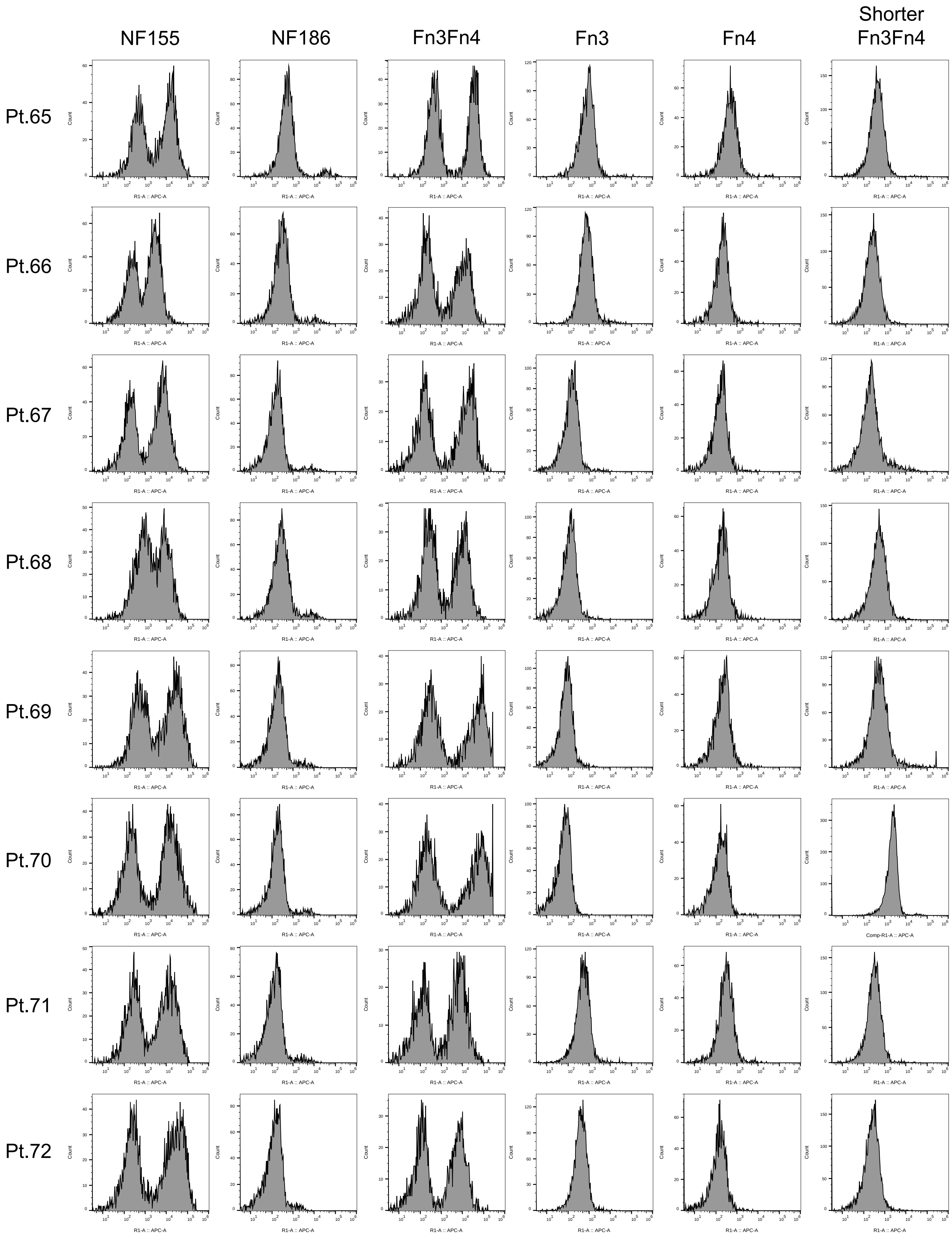
NF155
NF186
Fn3Fn4
Fn3
Fn4
Shorter Fn3Fn4
Pt.65
Pt.66
Pt.67
Pt.68
Pt.69
Pt.70
Pt.71
Pt.72
Count
R1-A :: APC-A
Comp-R1-A :: APC-A

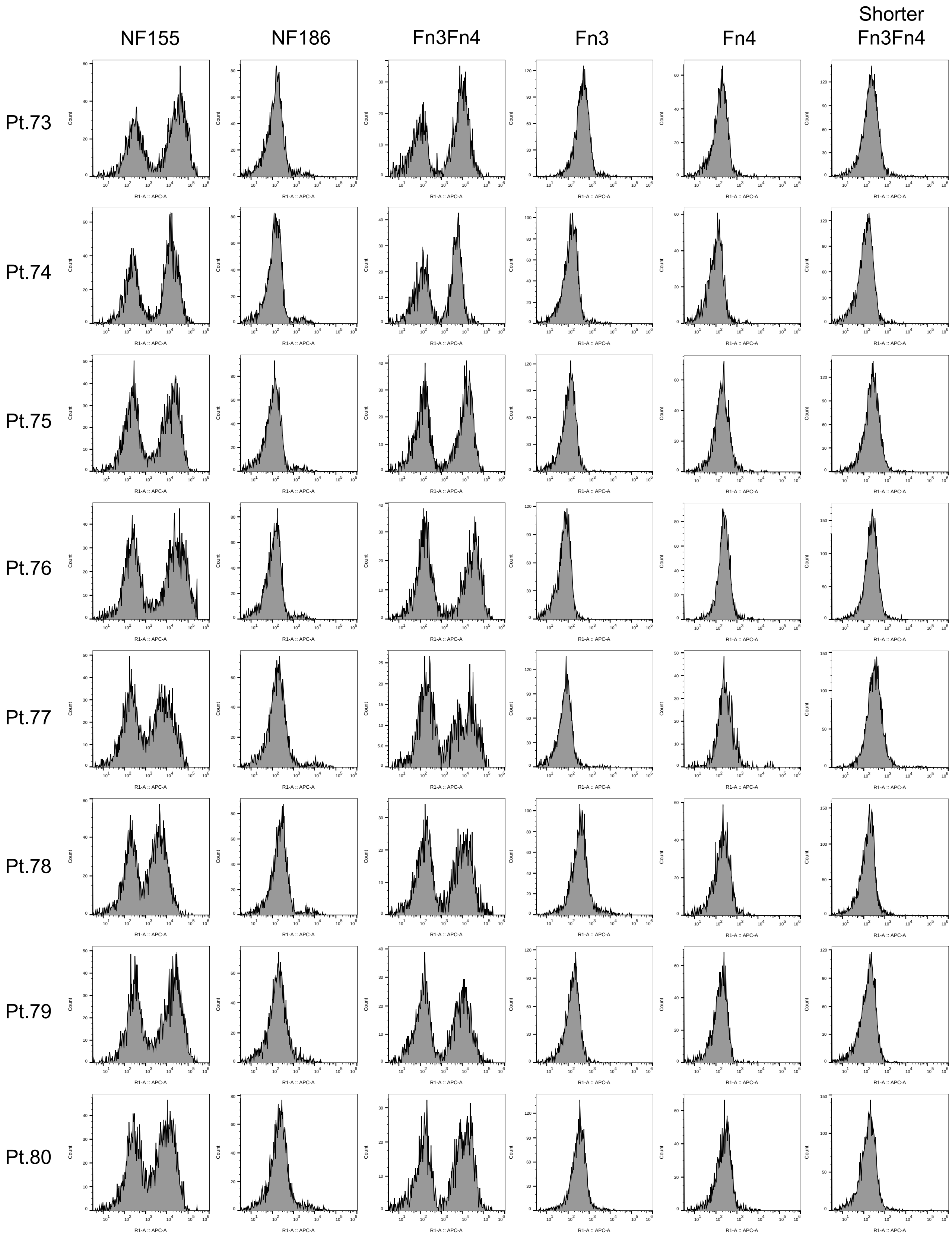
NF155
NF186
Fn3Fn4
Fn3
Fn4
Shorter Fn3Fn4
Pt.73
Pt.74
Pt.75
Pt.76
Pt.77
Pt.78
Pt.79
Pt.80
Count
R1-A :: APC-A

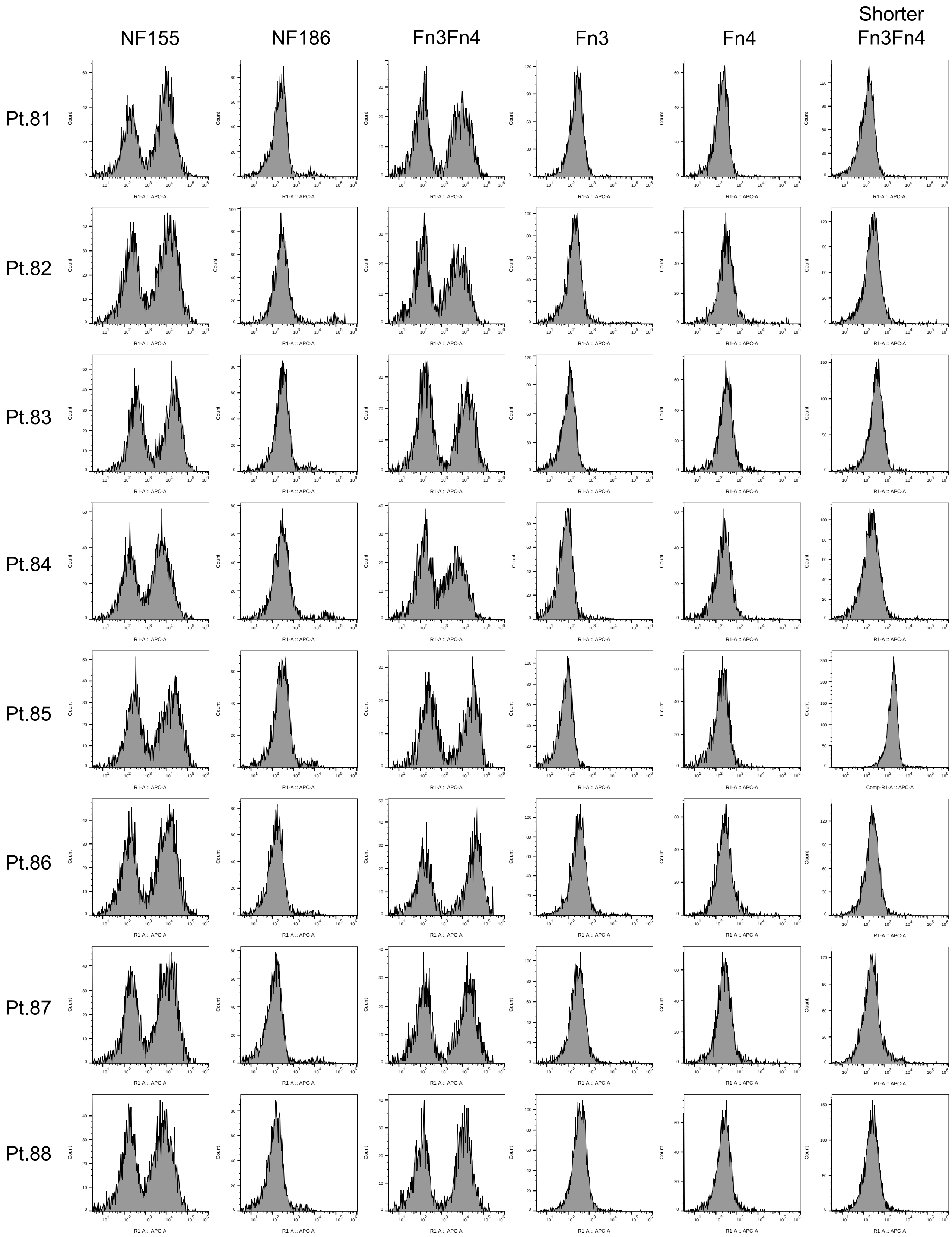
NF155
NF186
Fn3Fn4
Fn3
Fn4
Shorter Fn3Fn4
Pt.81
Pt.82
Pt.83
Pt.84
Pt.85
Pt.86
Pt.87
Pt.88
Count
R1-A :: APC-A
Comp-R1-A :: APC-A

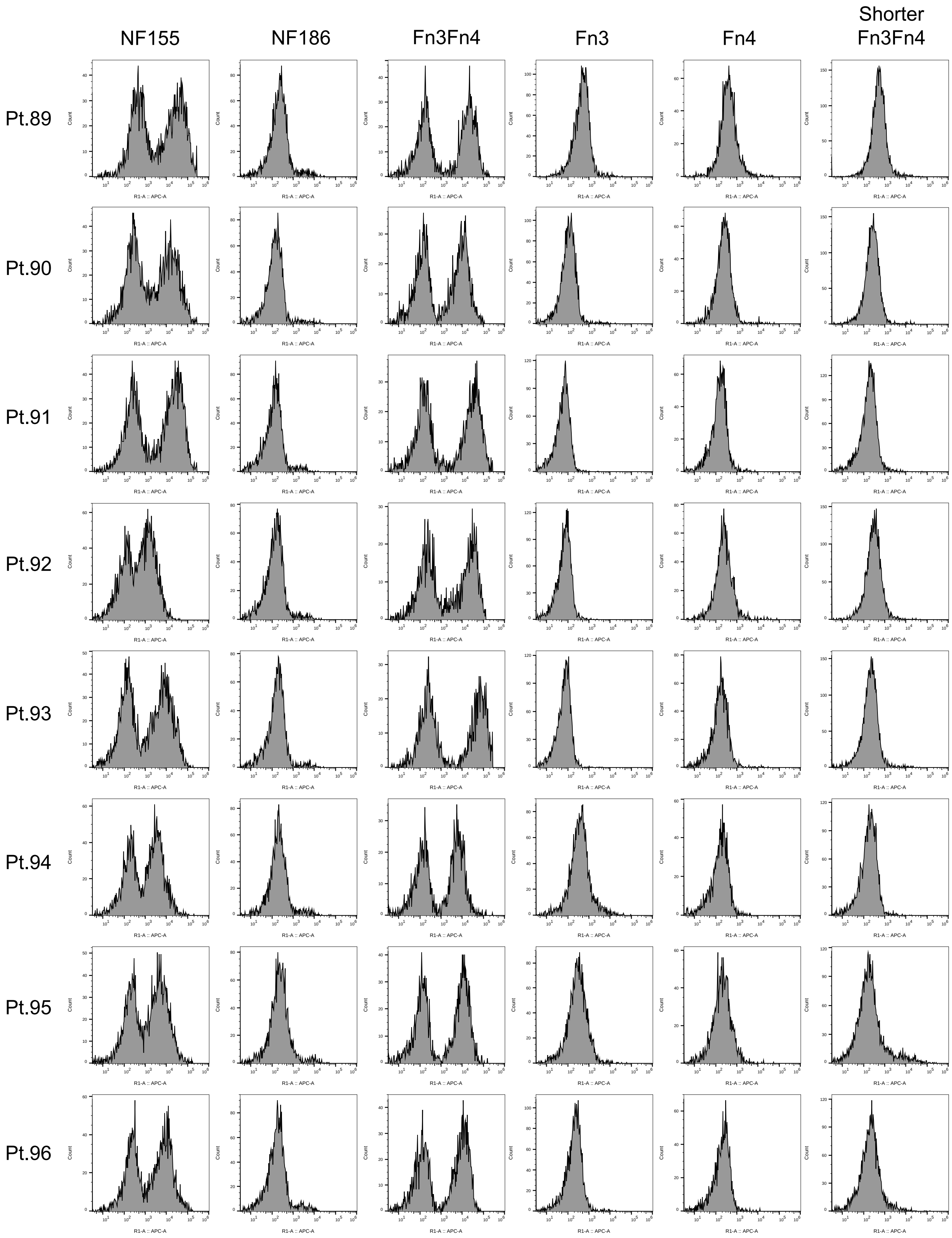
NF155
NF186
Fn3Fn4
Fn3
Fn4
Shorter Fn3Fn4
Pt.89
Pt.90
Pt.91
Pt.92
Pt.93
Pt.94
Pt.95
Pt.96
Count
R1-A :: APC-A

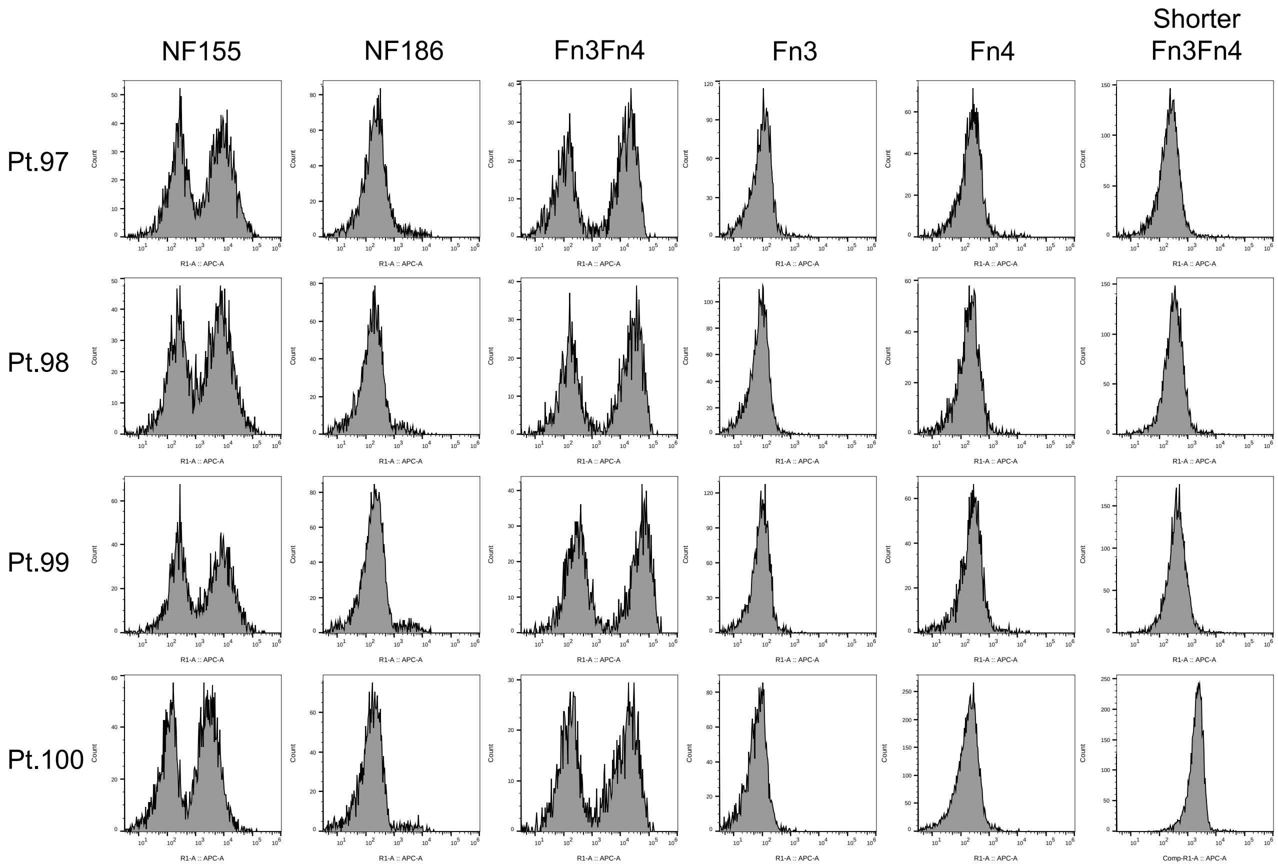
NF155
NF186
Fn3Fn4
Fn3
Fn4
Shorter Fn3Fn4
Pt.97
Pt.98
Pt.99
Pt.100
Count
R1-A :: APC-A
Comp-R1-A :: APC-A

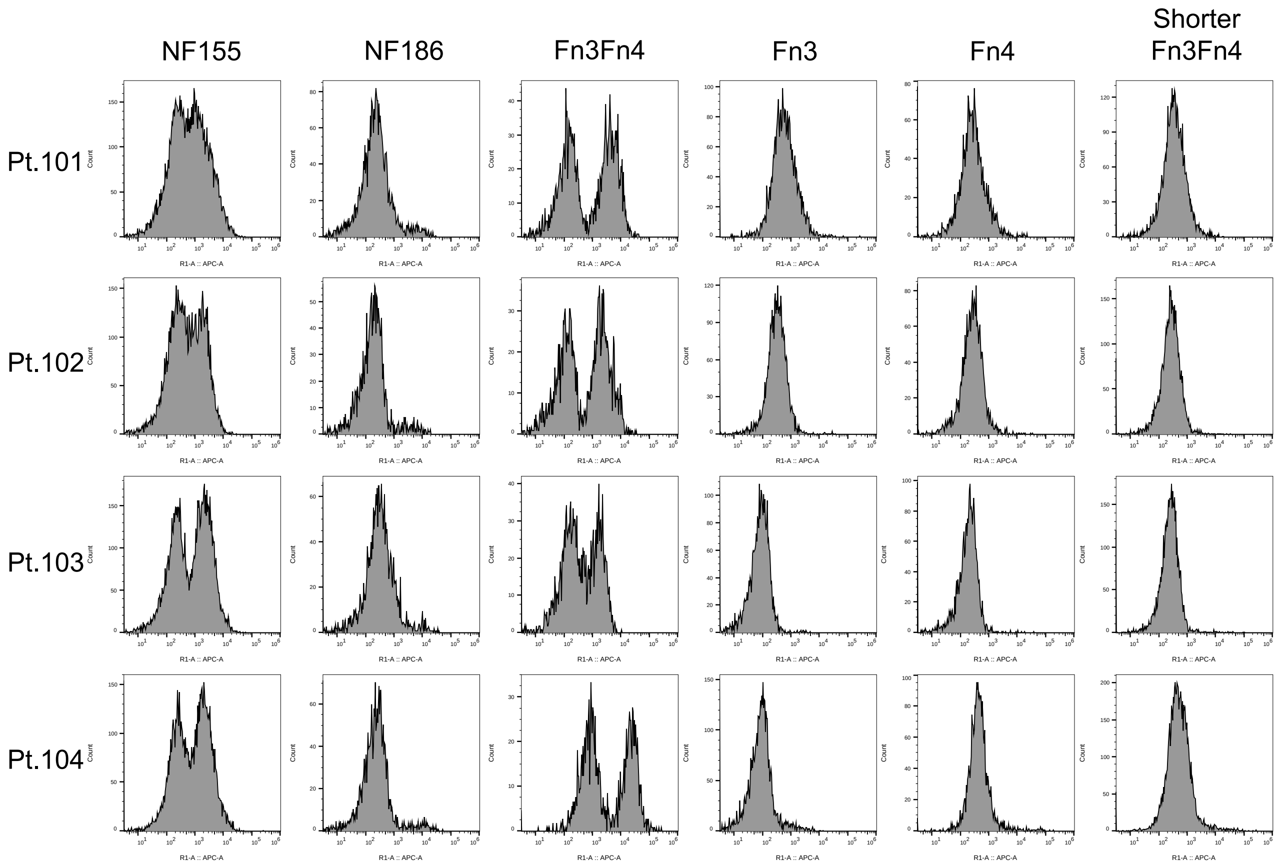

NF155
NF186
Fn3Fn4
Fn3
Fn4
Shorter Fn3Fn4
Pt.101
Pt.102
Pt.103
Pt.104
Count
R1-A :: APC-A
